# Self-Organized Memristive Ensembles of Nanoparticles Below the Percolation Threshold: Switching Dynamics and Phase Field Description

**DOI:** 10.3390/nano13142039

**Published:** 2023-07-10

**Authors:** Renat T. Sibatov, Andrey I. Savitskiy, Pavel E. L’vov, Yulia O. Vasilevskaya, Evgeny P. Kitsyuk

**Affiliations:** 1 Scientific-Manufacturing Complex “Technological Centre”, 124498 Moscow, Russia; 2 Department of Theoretical Physics, Moscow Institute of Physics and Technology (MIPT), 141700 Dolgoprudny, Russia; 3 Laboratory of Diffusion Processes, Ulyanovsk State University, 432017 Ulyanovsk, Russia; lvovpe@sv.uven.ru; 4 Institute of Integrated Electronics, National Research University of Electronic Technology (MIET), 124498 Moscow, Russia

**Keywords:** memristive networks, nanoparticles, percolation, synaptic plasticity

## Abstract

Percolative memristive networks based on self-organized ensembles of silver and gold nanoparticles are synthesized and investigated. Using cyclic voltammetry, pulse and step voltage excitations, we study switching between memristive and capacitive states below the percolation threshold. The resulting systems demonstrate scale-free (self-similar) temporal dynamics, long-term correlations, and synaptic plasticity. The observed plasticity can be manipulated in a controlled manner. The simplified stochastic model of resistance dynamics in memristive networks is testified. A phase field model based on the Cahn–Hilliard and Ginzburg–Landau equations is proposed to describe the dynamics of a self-organized network during the dissolution of filaments.

## 1. Introduction

To execute neural network algorithms with energy efficiency and interconnectivity comparable to the brain, it is necessary to emulate synaptic functionality [1,2]. There are attempts [3] to develop internally brain-like hardware architectures that could support neuromorphic computing in a more natural way than standard (highly organized) chip architectures. Self-organizing networks of nanoparticles and nanowires have recently become promising systems for neuromorphic computing [4,5,6]. These networks exhibit critical behavior near metal–insulator transition, scale-free networking that can provide new brain-like information processing with potentially attractive features such as ultra-low power consumption [7,8,9]. Below the percolation threshold, networks consist of groups of particles separated by tunnel gaps; the applied voltage causes the formation of atomic scale filaments in the gaps, and observed avalanches of switching events are similar to potentiation in biological neural systems [10]. In [9], such memristive nanosystems are called percolation with plasticity systems. The neuromorphic advantages of percolating nanomaterials with plasticity include multivalued memory, high dimensionality and non-linearity capable of transforming input data into spatiotemporal patterns, and no need for array interconnects [9].

Memristive devices demonstrate promising possibilities for data storage and processing, with advantages such as scalability, high speed, and compatibility with complementary metal–oxide–semiconductor (CMOS) technology [11,12]. Atomic-scale dynamics in memristive percolation networks mimics the “integrate-and-fire” mechanism with leakage peculiar to biological neurons [8]. Electrical responses from self-organized networks of nanoparticles exhibit spatiotemporal correlations and criticality of the percolation phase transition similar to those observed in the brain [10]. In particular, as is shown in [10], the sizes and durations of switching avalanches are distributed according to a power law, and the corresponding power law exponents satisfy strict criticality criteria. The avalanches in memristive percolation networks are quantitatively similar to the observed ones in cortical tissue [13], and they are characterized by correlations necessary for calculations. Further research is needed to better understand and validate avalanche criticality in memristive nanowire networks. In recent work [14], the dynamics of switching in a memristive network of silver nanoparticles coated with an insulating matrix is studied, and the authors point to the absence of qualitative differences in the critical dynamics and long-term correlations in networks of nanoparticles with and without a matrix. The use of dielectric coatings expands the possibilities of practical application of neuromorphic percolation networks. The insulating coating may increase the threshold voltages for switching to the memristive state [14].

It is important also to develop stochastic models to describe dynamics of conductance in memristive systems based on self-organized nanostructures. In recent work [15], a probabilistic model of random resistance jumps in memristive devices is proposed. In [16], a master equation is considered to analyze the networks composed of probabilistic binary memristors. The basic theory of percolation networks with plasticity is outlined in [9].

In the present work, percolative memristive networks based on self-organized ensembles of silver and gold nanoparticles are synthesized and investigated. Using cyclic voltammetry, pulse and step voltage excitations, we study switching between memristive and capacitive states. The observed plasticity can be manipulated in a controlled manner. The simplified stochastic model of resistance dynamics in memristive networks is testified. It is assumed that the main principle of operation of the considered memristive systems is the reconfiguration of conductive filaments in nanoscale switching gaps between nanoparticles in response to an applied external voltage. The process of formation of filaments in an ensemble of nanoparticles is reversible. According to simple thermodynamic principles, filaments dissolve over time [17]. In the last section, a phase-field model describing the dissolution of filaments on a substrate is presented.

## 2. Arrays of Silver and Gold Nanoparticles

**Figure 1:**
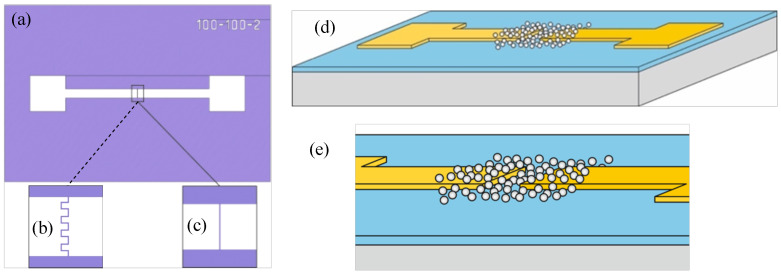
Sample topology (**a**) with interdigitated (**b**) and rectangular (**c**) electrode geometry; schematic view of nanoparticles array on electrodes in the interelectrode gap (**d**) with an enlarged image (**e**).

For electrophysical studies, two versions of planar structures with memristive networks were fabricated. Two Au electrodes of interdigitated and rectangular geometries are sputtered on a thermally oxidized silicon substrate with a SiO${}_{2}$ layer of thickness of H≈100 nm. The topologies of the structures are shown in Figure 1. In one implementation, the electrodes are of an interdigitated structure (Figure 1b) with pins 12 μm long and 10 μm wide. The gap between electrodes is 2 μm. In the other implementation, the electrodes are rectangular in shape (Figure 1c). The length of electrode tape is 800 μm, and the width is 100 μm. The gap between electrodes is 2 μm. Arrays of Ag or Au nanoparticles were formed on the surface in the gap between the electrodes (Figure 1d,e).

Ensembles of metal nanoparticles were formed by two-stage vacuum-thermal evaporation followed by heat treatment after each stage of evaporation. The size of particles and their density on the surface are determined by the amount of evaporated substance. The used method of nanoparticle formation is described in detail in [18,19]. The metal was deposited on the sample surface through a metal mask with a hole in the region of interelectrode gap, and the mask partially covered the electrodes.

**Figure 2:**
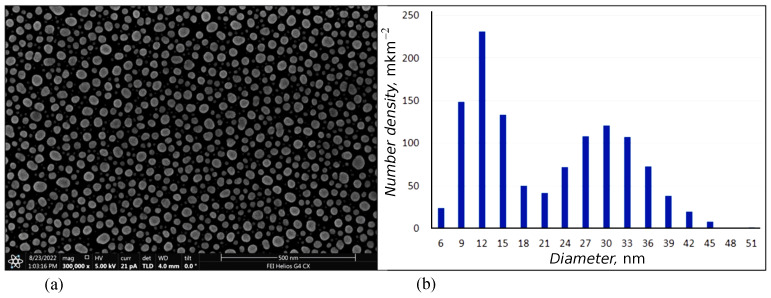
SEM image (**a**) and histogram of nanoparticle size distribution (**b**) for the array of Ag nanoparticles.

**Figure 3:**
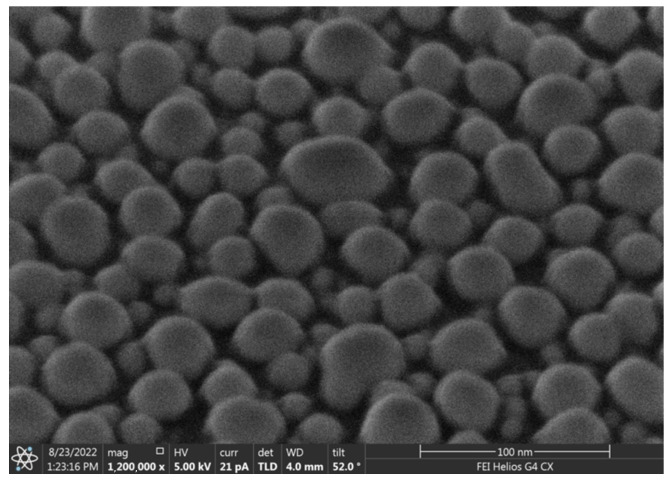
SEM image of an array of obtained Ag nanoparticles taken at an angle of 52°.

At the first stage, a larger weight portion of metal evaporated. After annealing the sample, an array of larger nanoparticles formed from the condensate on the crystal surface in the deposition region. The distance between nanoparticles in this method is directly proportional to the nanoparticle size [18,19]. To increase the particle density, a second stage of deposition was carried out. In this case, the evaporated weight portion of the metal was less than the previous one. After annealing, in the gaps between large nanoparticles formed at the first stage, small nanoparticles were formed, which led to a decrease in the distance between particles in the ensemble. Arrays of nanoparticles obtained in a two-stage process are characterized by a bimodal size distribution of nanoparticles. SEM images and a histogram of nanoparticle size distribution for the array of Ag nanoparticles are shown in Figure 2 and Figure 3.

To form arrays of Ag nanoparticles, weighed portions of metal with masses of 4.5 and 2.7 mg were evaporated; for Au, 11.3 and 6.8 mg were used. The distance from evaporator to deposition surface was 20 cm. Subsequent annealing after evaporation was carried out in vacuum at a temperature of 230 °C for arrays of Ag nanoparticles and 350 °C for arrays of Au nanoparticles. The annealing duration was 15 min. For all vacuum processes, the residual pressure did not exceed $10^{-5}$ Torr.

Depending on the annealing conditions, an array of Ag nanoparticles undergoes different transformations. The difference manifests itself in the value of predominant particle size and the type of particle size distribution. In the case of vacuum annealing, the distribution profile is narrower. Such an array of nanoparticles is characterized by a smaller spread of sizes relative to the predominant one. For this reason, in further experiments, during the formation of Ag and Au particles, annealings were carried out in vacuum. Further studies of the structures with ensembles of Ag and Au nanoparticles were carried out using an FEI Helios NanoLab 650i DualBeam scanning electron microscope and a Jeol JSM 6010 PLUS/LA scanning electron microscope.

**Figure 4:**
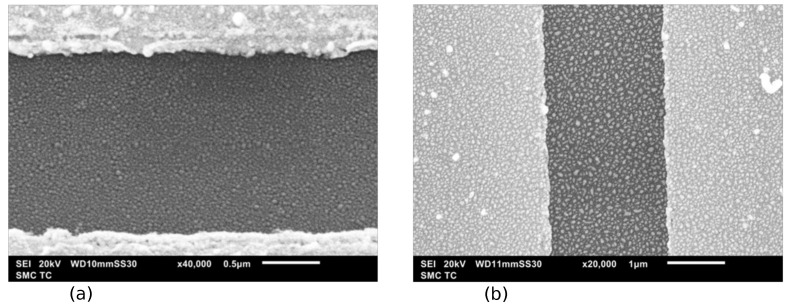
SEM image of the space between rectangular electrodes of a structure with an array of silver (**a**) and gold (**b**) nanoparticles.

**Figure 5:**
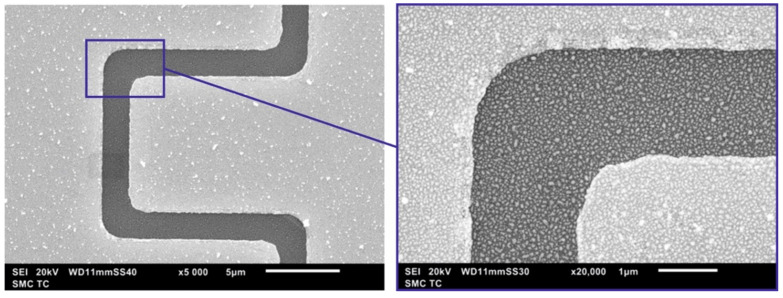
SEM image of the space between the electrodes of the interdigitated structure with an array of gold nanoparticles.

The resulting arrays of Ag and Au nanoparticles in the process of two-stage vacuum-thermal evaporation followed by heat treatment demonstrate a similar picture both in terms of the average particle size and the type of particle size distribution. Figure 4 and Figure 5 show the SEM images of a planar fragment of the structure with Ag NPs, as well as a histogram of the size distribution of NPs. The SEM images confirm the high density of particles per unit surface, which should facilitate the formation of filaments between particles.

## 3. Memristive Properties of Nanoparticle Ensembles

Percolative galvanoplastic networks of nanoparticles or nanowires are attractive for neuromorphic computations because they implement self-organized criticality characteristic of cortical tissue [20]. Experimental recordings of large groups of neurons showed bursts of activity, the so-called neural avalanches, with size and duration distributed according to power laws. There is a hypothesis that the collective dynamics of large neural networks in the brain operates near the critical point of the second-order phase transition (see [20] for details). Critical dynamics is also observed in percolation systems near the percolation threshold. The order parameter of the ‘metal–insulator’ phase transition is the conductance of a system, and the control parameter can be related to the probability *p* of current flow between neighboring nanoparticles. This probability is a dynamic variable because it depends on the formation of atomic-scale filaments between adjacent nanoparticles. In an infinite system, the phase transition takes place at the critical point $p=p_{c}$. In bounded systems, the phase transition occurs not at the critical point but in the critical region near $p_{c}$.

**Figure 6:**
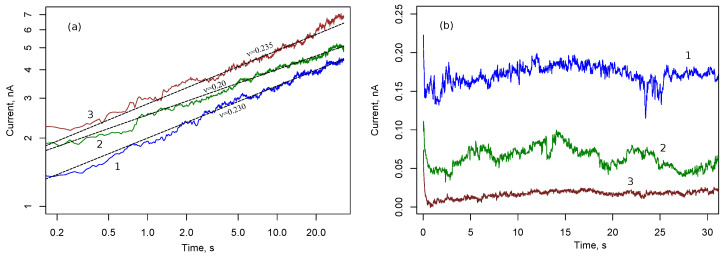
Current responses of cell 1 with rectangular electrodes and Ag nanoparticle array to voltage steps 20 V (**a**) and 5 V (**b**). Numbers 1, 2, and 3 correspond to three consecutive measurements with a silence interval of θ=30 s between them.

Before the stage of stable growth of filaments, the system of nanoparticles is insulating and can be characterized by a certain capacity. Switching between memristive and capacitive states was studied using cyclic voltammetry and excitation by step voltage. Figure 6 demonstrates the time dependencies of current in the array of silver NPs with rectangular electrodes after the application of constant voltage $U_{0}$. The duration of the single measurement is 30 s, then zero voltage was maintained for 2 min. After that, voltage $U_{0}$ is applied again. In Figure 6a, $U_{0}=20$ V and in Figure 6b $U_{0}=5$ V. Measurement data of three cycles are demonstrated. At a sufficiently high voltage $U_{0}$, a power law increase in current is observed, which corresponds to a similar dependence of conductance *G*. Power law exponent is of order 0.2.

An increase in conductivity is associated with a growing number of filaments between nanoparticles in the ensemble. In the case of $U_{0}=5$ V, the conductance dropped sharply, and the current decreases in successive cycles. This is apparently due to the destruction of filaments between the nanoparticles.

**Figure 7:**
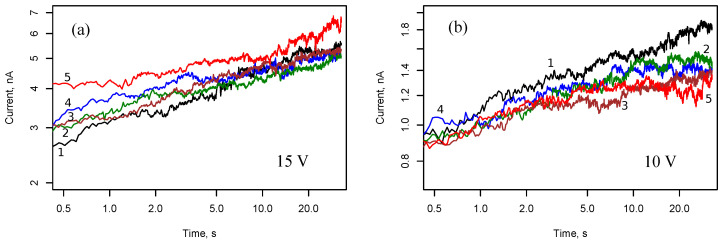
Current responses of cell 1 with rectangular electrodes and Ag nanoparticle array to voltage steps 15 V (**a**) and 10 V (**b**). Numbers 1, 2, 3, 4 and 5 correspond to five consecutive measurements with a silence interval of 30 s between them.

Similar time dependencies for $U_{0}=15$ V and $U_{0}=10$ V are shown in Figure 7. A power law trend persists for voltage $U_{0}=15$ V and becomes less noticeable for voltage $U_{0}=10$ V. The probability of filament formation is reduced, and conductance fluctuations are more pronounced at smaller voltages.

The small relative fluctuations at 20 V are apparently associated with the functioning of several conduction pathways and the self-averaging effect. For a voltage of 5 V, a stochastic dynamics of the conductance is observed, which corresponds to a stationary random process, and the average value of the conductance and its dispersion remain approximately constant. This means that when the voltage is reduced by a factor of 3–4, the configuration associated with a certain distribution of filaments remains the same on average; that is, the memristive state is preserved and it is supported by a lower voltage. In the absence of currents, the configuration with the formed filaments is highly nonequilibrial, and the system tends to reduce the surface energy due to the dissolution of filaments and distribution of substance over the surface of nanoparticles.

**Figure 8:**
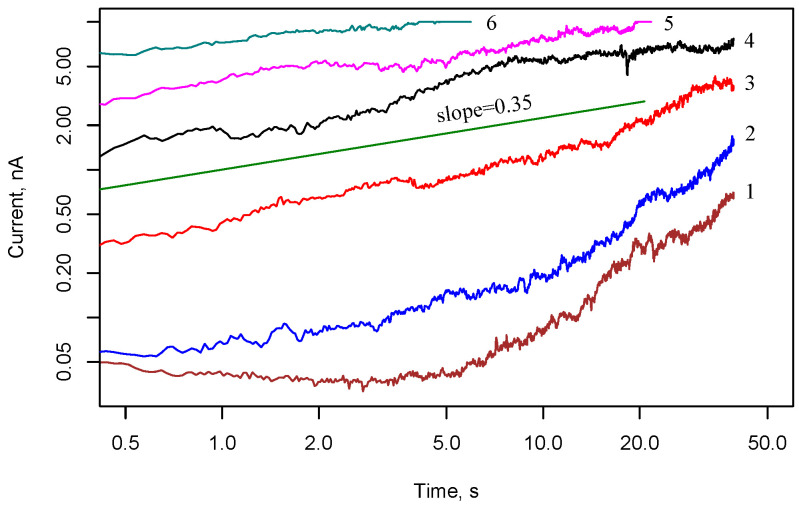
Current responses in cell 2 with interdigitated electrodes and Ag nanoparticle array to voltage steps 10 V. Numbers 1–6 correspond to six consecutive measurements with a silence interval of 30 s between them.

Figure 8 demonstrates the current in cell 2 with interdigitated electrodes and Ag nanoparticle array after applying a voltage step of 10 V. In general, the observed dynamics in the case of interdigital electrode geometry is similar to the case of rectangular electrodes. However, one can note a decrease in the voltage for switching to the memristive state.

At a low voltage, the field is distributed in a chaotic manner, the dynamic voltages between nanoparticles are insufficient for the formation of filaments, and the formed atomic bridges are predominantly destroyed. The transition to the memristive state is associated with the formation of a percolation cluster connecting two electrodes, but this cluster itself is apparently unstable and is capable of dynamically rearranging with voltage variations. It should be noted that the processes of tunneling between nanoparticles are of great importance during current flow. The observed current values correspond to conductivities less than the conductance quantum $G_{0}=2e^{2}/h\approx 7.748\times 10^{-5}$ S. This means that the connectivity parameter is still below the percolation threshold.

**Figure 9:**
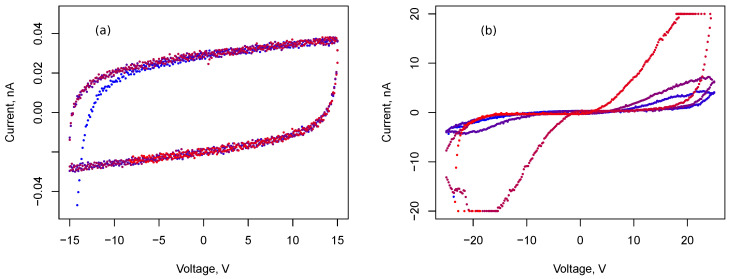
Cyclic voltammograms of a two-terminal system with rectangular electrodes and an array of silver nanoparticles. The voltammograms correspond to three cycles. Blue points correspond to the beginning of measurement, and red color points correspond to the end of measurement. Here, ΔU=15 V (**a**) and ΔU=25 V (**b**).

**Figure 10:**
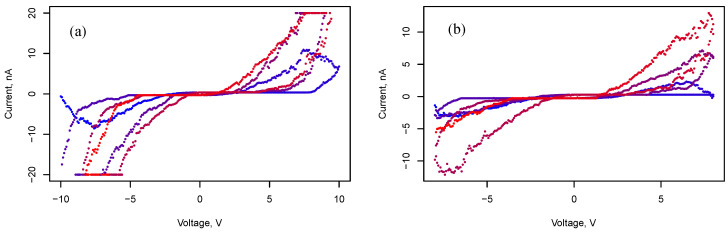
Cyclic voltammograms of a two-terminal system with interdigital electrodes and an array of silver nanoparticles. Blue points correspond to the beginning of measurement, and red color points correspond to the end of measurement. Here, ΔU=10 V (**a**) and ΔU=8 V (**b**).

**Figure 11:**
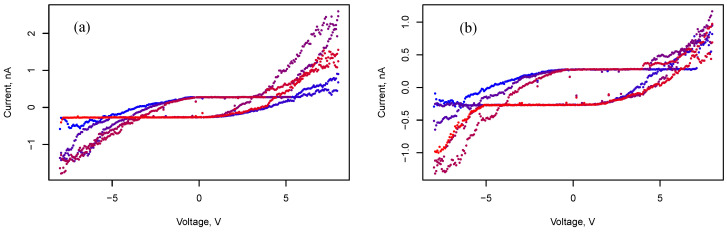
Cyclic voltammograms of a two-terminal system with interdigital electrodes and an array of silver nanoparticles. Blue points correspond to the beginning of measurement, and red color points correspond to the end of measurement. Here, ΔU=8 V (**a**) and ΔU=8 V (**b**), after θ=30 s.

Staying in the capacitive mode and switching to the memristive state with voltage variation is shown in Figure 9, Figure 10 and Figure 11. Figure 9 demonstrates cyclic voltammograms with different voltage amplitudes ΔU: 15 V, and 25 V. The duration of a cycle is Δt = 30 s. Memristive behavior is observed for interdigital behavior as well (Figure 10 and Figure 11). Moreover, the voltammograms (Figure 11) demonstrate an intermediate state between memristive and capacitive behavior. Such an intermediate state persists for a relatively long time. A series of measurements of voltammograms with an amplitude of 8 V and duration of 3 min was carried out at three cycles per measurement. During 10 such measurements in a series, the system retained this intermediate state; when the amplitude decreased by 0.5 V, the system switched to a capacitive state.

In [21], Bai Sun et al. proposed a physical model of a memristor connected in parallel with a capacitor. This model explains how the nonpinched hysteresis behavior of IV curves originates from the capacitive-coupled memristive effect. A similar effect is clearly visible from the voltammograms shown in Figure 11. It is noteworthy that the capacitance determined from the measurements presented in Figure 9 turns out to be 39.6 pF in the memristive state versus 5.5 pF in the simple capacitive state. This is apparently due to the presence of stable filaments in the memristive state, which lead to a decrease in the distance between charged regions and the involvement of new clusters of nanoparticles in the formation of capacitance.

**Figure 12:**
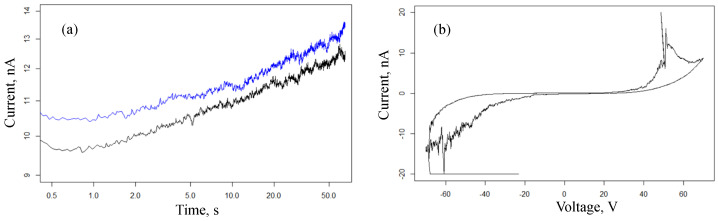
Current response (60 V) (**a**) and cyclic voltamogramm ΔU=65 V (**b**) for cell 3 with interdigital electrodes and gold (Au) nanoparticle array. Black and blue curves in plot (**a**) correspond to two consecutive measurements with a silence interval of 30 s between them.

Current responses and the voltammogram for cell 3 with interdigital electrodes and a gold (Au) nanoparticle array are given in Figure 12. It can be seen from these graphs that for an array of gold nanoparticles, we observe a similar behavior: a power law increase in conductance at a constant voltage, switching to a memristive state. However, the latter occurs at a significantly higher voltage of the order of 50 V.

## 4. Modeling of Resistance Dynamics

In recent work [15], a probabilistic model of random resistance jumps in memristive devices is proposed. In [16], a master equation is considered to analyze the networks composed of probabilistic binary memristors. The basic theory of percolation networks with plasticity is outlined in [9].

To explain the dynamics of conduction in our arrays of nanoparticles, we invoke ideas previously used to explain the kinetics of transient current in arrays of colloidal semiconductor nanocrystals [22,23]. In arrays of nanoparticles, the current decay after application of step voltage $U\left(t\right)=U_{0}l\left(t\right)$ is often described by power law [24,25]:

(1) $$I\left(t\right)\propto t^{-\alpha },\;0<\alpha <1,$$

Here l(t) is the Heaviside step function. The exponent α is less than 1, and in the general case, its value depends on the nanoparticle size and temperature. In [25], the authors point out that the current (1) is not a bias current, because the integral of Equation (1) is associated with the charge flowing through the sample $Q=\int_{0}^{\infty }I\left(t\right)dt\to \infty$. Below the percolation threshold, the current in our array of nanoparticles is controlled by tunneling processes between isolated clusters of nanoparticles in channels with the lowest integral tunneling resistance. It is assumed that filaments have not yet formed between these clusters. Moreover, we assume that the characteristic time of pulse propagation is much shorter than the characteristic time of evolution of filaments between nanoparticles.

The observed non-exponential relaxation of current can be explained by the time dependence of the state of the system. Ref. [26] proposed decreasing the charge flow due to the suppression of injection from the contact. This suppression arises because electrons trapped in a nanocrystal prevent the transport of other electrons through this nanocrystal, and the flow is jammed. Morgan et al. [24] explain the power law decay of current I(t) by the presentation of non-equilibrium electrons distributed over a nanocrystal array as the Coulomb glass.

Novikov et al. [25] proposed the model utilizing the Lévy statistics. In their model, an array of nanoparticles consists of N≫1 identical independent conduction channels operating in the parallel regime. Each channel opens at random times and conducts a current pulse. Novikov et al. [25] assumed that these channels are characterized by the distribution of waiting times *T* between successful current pulses. In addition, they postulated that this distribution is a heavy-tailed power law [25],

(2) $$\Psi \left(t\right)=P\left(T>t\right)\propto t^{-\nu },\;0<\nu <1,\;t\to \infty .$$

Such a heavy-tailed distribution leads to the divergence of the mean value; it provides specific statistical properties of the process, particularly, the presence of memory effects [22].

The distribution of the number of pulses in a channel is

$$p_{n}=P\left(N\left(t\right)=n\right)=P\left(N\left(t\right)<n+1\right)-P\left(N\left(t\right)<n\right)=P\left(T_{n+1}>t\right)-P\left(T_{n}>t\right),$$

where $T_{n}=\sum_{i=0}^{n}T_{i}$. According to the generalized limit theorem (see e.g., [27]),

$$P\left(T_{n}<t\right)∼G_{+}\left(cn^{-1/\nu }t;\nu \right),\;t\to \infty .$$

Here, $G_{+}\left(t;\nu \right)$ is the one-sided Lévy stable distribution function. Thus, we have

$$p_{n}∼G_{+}\left(cn^{-1/\nu }t;\nu \right)-G_{+}\left(c{\left(n+1\right)}^{-1/\nu }t;\nu \right)∼\nu^{-1}n^{-1-1/\nu }ct\;g_{+}\left(cn^{-1/\nu }t;\nu \right),$$

where $g_{+}\left(t;\nu \right)$ is the Lévy stable density. The current is determined by the expression

$$i\left(t\right)=\frac{d\langle Q\rangle }{dt}=eZ\frac{d}{dt}\sum np_{n}∼eZ\nu c{\left(ct\right)}^{\nu -1}\int_{0}^{\infty }s^{-\nu }g_{+}\left(s;\nu \right)ds=\frac{eZc^{\nu }}{\Gamma (\nu )}\;t^{\nu -1},$$

for $t\gg c^{-1},\;0<\nu <1.$ Here, *Z* is the number of channels.

Exponent α of power law decay is related to the model parameter ν by relation α=1−ν. Current (1) presents the response to a voltage step, so more general voltage signal can be presented in the superposition form of superposition of steps: $u\left(t\right)\approx \sum_{i}\Delta u_{i}\;l\left(t-i\Delta t\right)$. Consequently, we have

$$i\left(t\right)\propto \lim_{\Delta t\to 0}\sum_{i}\Delta u_{i}{\left(t-t_{i}\right)}^{-\alpha }=\frac{d}{dt}\int_{0}^{t}\frac{u(t^{\prime })}{{\left(t-t^{\prime }\right)}^{\alpha }}dt^{\prime }.$$

It is known that the operator

$${}_{0}D_{t}^{\alpha }u\left(t\right)=\frac{1}{\Gamma (1-\alpha )}\frac{d}{dt}\int_{0}^{t}\frac{u(t^{\prime })}{{\left(t-t^{\prime }\right)}^{\alpha }}dt^{\prime }$$

is the fractional (0<α<1) Riemann–Liouville derivative. Note that the initial time moment t=0 implies that u=0 in the interval (−∞,0). In a more general case,

(3) $$i\left(t\right)=K_{\alpha }\;{}_{-\infty }D_{t}^{\alpha }u\left(t\right).$$

When α→0, this relation represents the Ohm law for a conductor with conductivity $K_{0}$; when α→1, the relation coincides with the expression for an ideal dielectric with capacity $K_{1}$. In a more general case, the dynamics seems to be described by an equation of variable order. The dependence of the order on time and current can provide the memristive properties of the system.

The large spread of resistances between neighboring nanoparticles is confirmed by first-principles calculations. Due to the exponential sensitivity of the tunneling probability to the distance between neighboring nanoparticles, fluctuations in the positions of atoms in the gap between nanoparticles lead to a very wide distribution of resistance and conductance.

Using the QuantumATK S-2021.06 software, the simulation of electronic conduction between silver nanoelectrodes is performed.The system simulating the device is divided into three regions (left electrode, central part, and right electrode). The implementation is based on the screening approximation. Within this approximation, it is assumed that the properties of the left and right electrodes are described by solving the problem for periodic electrode cells. The approximation is valid when the current through a system is small enough that the electrodes can be characterized by an equilibrium distribution of electrons.

In QuantumATK, the transfer matrix is calculated according to the following formula

$$\tau_{nm}\left(E,k\right)=\sum_{\ell }t_{n\ell }\left(E,k\right)t_{\ell m}^{†}\left(E,k\right),$$

where $t_{nk}$ is the transfer amplitude from the Bloch state $\psi_{n}$ in the left electrode to the Bloch state $\psi_{k}$ in the right electrode. The matrix $t^{†}$ is a Hermitian conjugate. The transmittance is defined as the trace of the transmission matrix,

$$\tau \left(E,k\right)=\sum_{n}\tau_{nn}\left(E,k\right).$$

Let $\lambda_{\alpha }$ be the eigenvalues of the transfer matrix $\tau_{nm}$. From the invariance of the trace of the matrix:

$$\tau \left(E,k\right)=\sum_{\alpha }\lambda_{\alpha }\left(E,k\right),$$

where $\lambda_{\alpha }\in \left[0,1\right]$ are transmission eigenvalues for each spin channel.

The transmission eigenstates are calculated by diagonalizing a linear combination of Bloch states, $\sum_{n}e_{\alpha ,n}\psi_{n}$, where $e_{\alpha ,n}$ are vectors of the basis diagonalizing the transmission matrix:

$$\sum_{m}\tau_{nm}e_{\alpha ,m}=\lambda_{\alpha }e_{\alpha ,n}.$$

**Figure 13:**
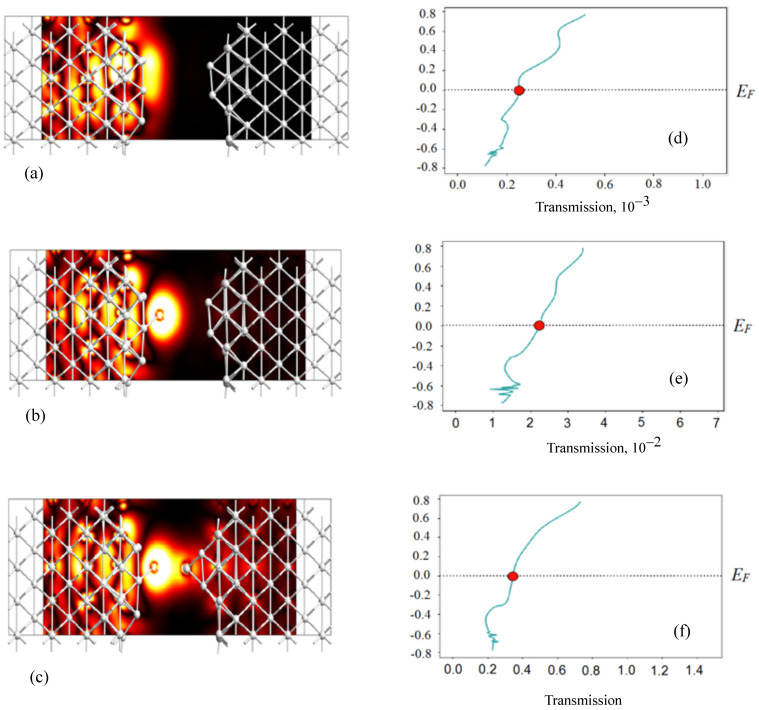
Left: thermal diagram of eigenstates corresponding to transmission coefficients at the Fermi energy level, at a distance of 9.17 Å (**a**), with the addition of an atom from one (**b**) and two sides (**c**). The amplitude scale on the heat map is from 0 (minimum—black) to 0.2 eV${}^{-1}/2$Å${}^{-3/2}$ (maximum—white); Right: corresponding transmission spectra; transmission values at Fermi energy level: τ=0.000253 (**d**), τ=0.0225 (**e**), τ=0.347 (**f**).

Figure 13 demonstrates examples of transmission eigenstates. We used the PseudoDojo Linear Combination of Atomic Orbitals (LCAO) pseudopotential. The exchange-correlation potential was described by the generalized gradient approximation (GGA) with the Perdew–Burke–Ernzerhof (PBE) functional. The density cut-off grid was 105 Ha (1 Ha = 27.21 eV). The Monkhorst–Pack method was used to generate points in the Brillouin zone. For the simulated system, periodic boundary conditions were set in the transverse direction; the two outer layers were fixed. The distance between the boundary atoms in the gap between the nanoparticles is 9.17 Å (Figure 13a). An atom was added on one side (Figure 13b) and on the other side (Figure 13c), a significant change in the conductance of the system is observed. Transmission values at the Fermi energy level are τ=0.000253 (Figure 13d), τ=0.0225 (Figure 13e), and τ=0.347 (Figure 13f). The corresponding conductance values are found by multiplying by the conductance quantum $G_{0}=2e^{2}/h$.

**Figure 14:**
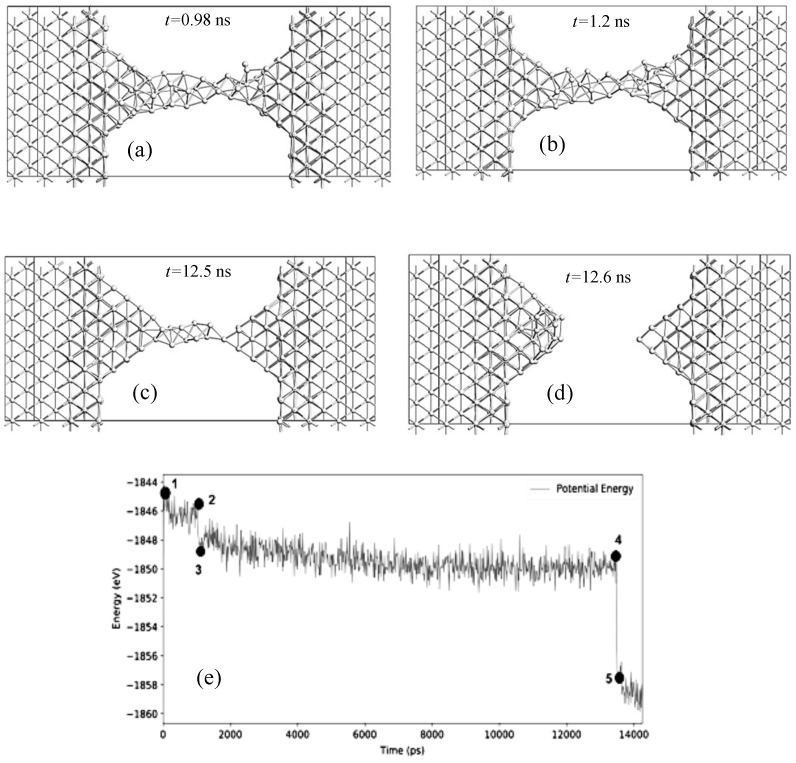
Simulation of the temporal stability of silver nanofilaments at 300 K (nanowire length ≈26 Å, width ≈7.2 Å): after 0.98 ns (**a**), 1.2 ns (**b**), 12.5 ns (**c**), 12.6 ns (**d**). The change in energy over time is shown in plot (**e**), the breakage of filament is accompanied by a sharp increase in the energy modulus.

In the calculations of molecular dynamics, we used the interaction potential from [28] calculated by the embedded atom method. A typical dynamics of filament dissolution in simulated systems can be represented as the following stages (Figure 14): (1) at the initial stage, the shape of the nanowire changes due to thermal vibrations of the system; (2) the nanowire loses its crystallinity, and the atoms in the filament begin randomly walking and can pass to the nanoparticles; (3) broadened protrusions are formed at the filament bases that facilitate the transfer of atoms to nanoparticles; (4) when the filament becomes sufficiently narrow, mainly in the middle, a break occurs, and then, the protrusions spread diffusively over the surface of nanoparticles.

Recent results of molecular dynamics modeling of filaments, including taking into account the graphite substrate, are given in the works [17,29]. The formation of sufficiently wide filaments during the functioning of the memristive network can contribute to the subsequent coalescence of nanoparticles, i.e., the nanobridge does not break: it pulls the nanoparticles together [30]. Undoubtedly, the substrate on which the nanoparticles are located significantly affects the migration of atoms between nanoparticles, the distribution of the electric field, and, consequently, the processes of the formation and dissolution of filaments. Filaments dissolve much more slowly in the presence of a substrate; therefore, other approaches such as Monte-Carlo algorithms or phase-field models turn out to be useful, which not only extend the time range but also allow one to simulate the dynamics of filaments in sufficiently large ensembles of nanoparticles. In the next section, we propose a phase-field model based on the Cahn–Hilliard and Ginzburg–Landau equations.

## 5. Phase Field Model of Thermodynamic Stability of Filaments

Let us consider the problem of filament ensemble stability in a two-dimensional model system using phase field theory. The system consists of the crystallized particles, which can be interconnected by filaments, thus forming a percolation structure. These filaments are prevously formed by the applied electric field or other factors. The free energy density functional *G* of the system reads

(4) $$G=n_{0}\int_{S}\left(g\left(c,\eta \right)+\frac{1}{2}\kappa_{c}{\left(\nabla c\right)}^{2}+\frac{1}{2}\kappa_{\eta }{\left(\nabla \eta \right)}^{2}\right)dA,$$

where $n_{0}$ is the number of atoms per unit surface of the substrate, g(c,η) is the free energy density per particle, $c\equiv c(\vec{r},t)$ is the concentration field of atoms, which can be both in the crystallized phase and form filaments connecting particles, $\eta \equiv \eta (\vec{r},t)$ is the order parameter that determines the distribution of crystallized particles on the substrate, dA is the substrate surface element, *S* is the total substrate surface area, and $\kappa_{c}$ and $\kappa_{\eta }$ are the gradient energy coefficients. The atomic concentration *c* and the order parameter η change from zero to one. Here, η=1 and c∼1 correspond to crystallized particles, η=0 and c∼0 correspond to the matrix, and η=0 and c∼1 correspond to filaments or formed particles in the amorphous state.

The dynamics of the concentration field and the order parameter can be obtained using the Cahn–Hilliard and Ginzburg–Landau equations:

(5) $$\begin{array}{c} \frac{\partial c}{\partial t}=n_{0}M\nabla^{2}\left[\frac{\partial g}{\partial c}-\nabla \cdot \left\{\kappa_{c}\nabla c\right\}\right]+\nabla \cdot {\vec{\xi }}_{c}, \end{array}$$

(6) $$\begin{array}{c} \frac{\partial c}{\partial t}=n_{0}L\left[\kappa_{\eta }\nabla^{2}\eta -\frac{\partial g}{\partial \eta }\right]+\xi_{\eta }. \end{array}$$

Here, we introduce the designation of the mobilities *M* and *L*, which have constant values, and also take into account the presence of fluctuations ${\vec{\xi }}_{c}$ and $\xi_{\eta }$ of the concentration field and the order parameter [31,32,33,34,35].

Assuming that the parameters of interaction between atoms inside the particles and in the matrix are different, we can write the free energy density in the form:

(7) $$g\left(c,\eta \right)=g^{P}\left(c\right)h\left(\eta \right)+g^{M}\left(c\right)\left[1-h(\eta )\right]+\frac{1}{2}W\eta^{2}{\left(1-\eta \right)}^{2},$$

where *W* is the interaction parameter, and $g^{P}$ and $g^{M}$ are the free energy densities in particles and the matrix, which can be determined in the regular solution approximation:

(8) $$\begin{array}{ccc} g^{P}\left(c\right) & = & g^{P}c+\Omega^{P}c\left(1-c\right)+k_{B}T\left(c\ln c+(1-c)\ln(1-c)\right), \end{array}$$

(9) $$\begin{array}{ccc} g^{M}\left(c\right) & = & g^{M}c+\Omega^{M}c\left(1-c\right)+k_{B}T\left(c\ln c+(1-c)\ln(1-c)\right), \end{array}$$

The interaction parameters $g^{P}$, $g^{M}$, $\Omega^{P}$ and $\Omega^{M}$ can be easily related to the interatomic interaction energies of solutions [36,37], $k_{B}$ is the Boltzmann constant, and *T* is the temperature. The approximating function h(η) can be chosen as follows: $h\left(\eta \right)=\eta^{2}\left(3-2\eta \right)$ [38,39]. Taking into account expressions (8) and (9), the free energy density (7) is expressed as

(10) $$\begin{array}{c} g\left(c,\eta \right)=\left(g^{P}h\left(\eta \right)+g^{M}\left(1-h\left(\eta \right)\right)\right)c+\left(\Omega^{P}h\left(\eta \right)+\Omega^{M}\left[1-h(\eta )\right]\right)c\left(1-c\right)+\\ k_{B}T\left(c\ln c+(1-c)\ln(1-c)\right)+\frac{1}{2}W\eta^{2}{\left(1-\eta \right)}^{2}. \end{array}$$

Since the gradient energy coefficient in binary systems is proportional to the interaction parameter ($\kappa^{M,P}∼\Omega^{M,P}$) [40,41], a similar approximating dependence on the order parameter can be used $\kappa \left(\eta \right)=\kappa^{P}h\left(\eta \right)+\kappa^{M}\left(1-h\left(\eta \right)\right)$.

The random fields $\xi_{c}$ and $\xi_{\eta }$ introduced in Equations (5) and (6) determine thermal fluctuations, and they can be specified using the correlation functions:

(11) $$\begin{array}{ccc} \langle \xi_{ci}\left(\vec{r},t\right)\xi_{cj}\left(\vec{r}{\;}^{\prime },t^{\prime }\right)\rangle & = & 2k_{B}TM\delta_{ij}w(|\vec{r}-\vec{r}{\;}^{\prime }\left|\right)\delta \left(t-t^{\prime }\right),\\ \langle \xi_{\eta }\left(\vec{r},t\right)\xi_{\eta }\left(\vec{r}{\;}^{\prime },t^{\prime }\right)\rangle & = & 2k_{B}TLw(|\vec{r}-\vec{r}{\;}^{\prime }\left|\right)\delta \left(t-t^{\prime }\right), \end{array}$$

where $\delta_{ij}$ is the Kronecker symbol and δ(t) is the Dirac delta function. As the function $w(\vec{r}-\vec{r}{\;}^{\prime })$, which determines the spatial correlation of the concentration field and the order parameter, we use the Gaussian function [33]:

(12) $$w(|\vec{r}-\vec{r}{\;}^{\prime }\left|\right)=\frac{1}{{\left(\sqrt{2\pi }\lambda \right)}^{d}}\exp\left[\frac{-|\vec{r}-\vec{r}{\;}^{\prime }{|}^{2}}{2\lambda^{2}}\right],$$

λ is the correlation length of fluctuations, and *d* is the dimension of the system (d=2).

Assuming that the mobility *M* and *L* are constant, it is convenient to pass to the following units of physical quantities used in Equations (5) and (6):

(13) $$\begin{array}{c} \left[\vec{r}\right]=l,\left[g^{P,M}\right]=\left[W\right]=\left[\Omega^{P,M}\right]=\Omega^{M},\left[T\right]=T_{C},\\ \left[t\right]=n_{0}M\Omega^{M}/l^{2},\left[\kappa_{\eta }\right]=\left[\kappa_{c}\right]=\Omega^{M}l^{2},\left[L\right]=M/l^{2}, \end{array}$$

where *l* is the characteristic length, which can be taken as equal to several interatomic distances, and $T_{C}=\Omega^{M}/2k_{B}$ is the critical temperature in the matrix.

**Figure 15:**
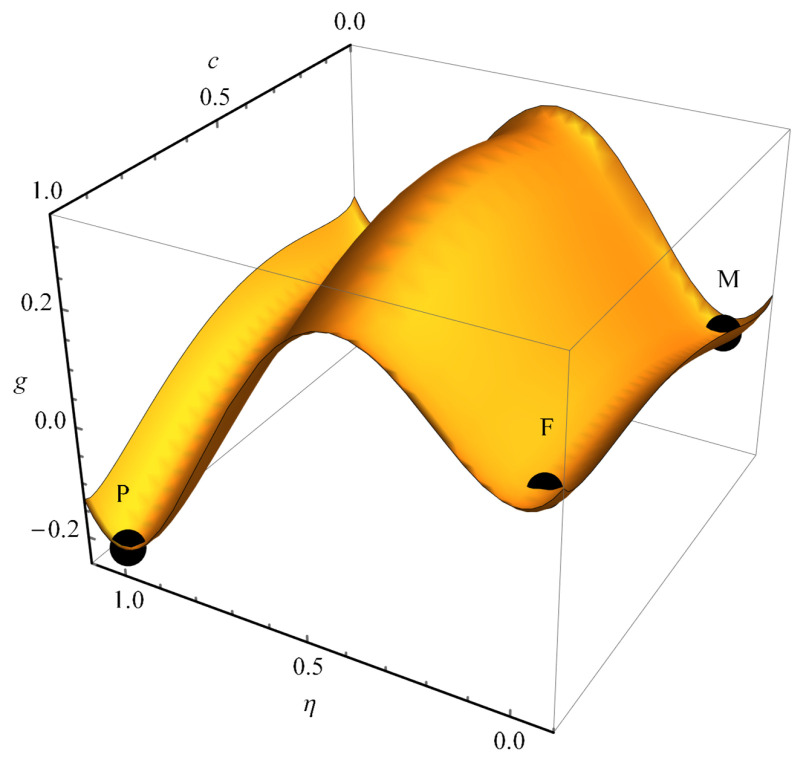
The dependence of free energy density on order parameter and concentration for the interaction parameters from Table 1.

Further modeling will be carried out for the system parameters given in Table 1. The dependence of the free energy density on the value of concentration *c* and order parameter η is shown in Figure 15. This dependence is characterized by three minima that determine the stable states of the system, which correspond to a homogeneous matrix without filaments (M), a matrix with a filament or non-crystallized nucleus (F), and a solute-enriched crystallized particle (P), which is energetically the most favorable. Transitions of particles between given stable states are characterized by barriers, the height of which determines the probabilities of transition between them. In this regard, it is expected that filaments (F), which are characterized by higher energy than particles (P), correspond to metastable formations and will gradually disappear.

**Figure 16:**
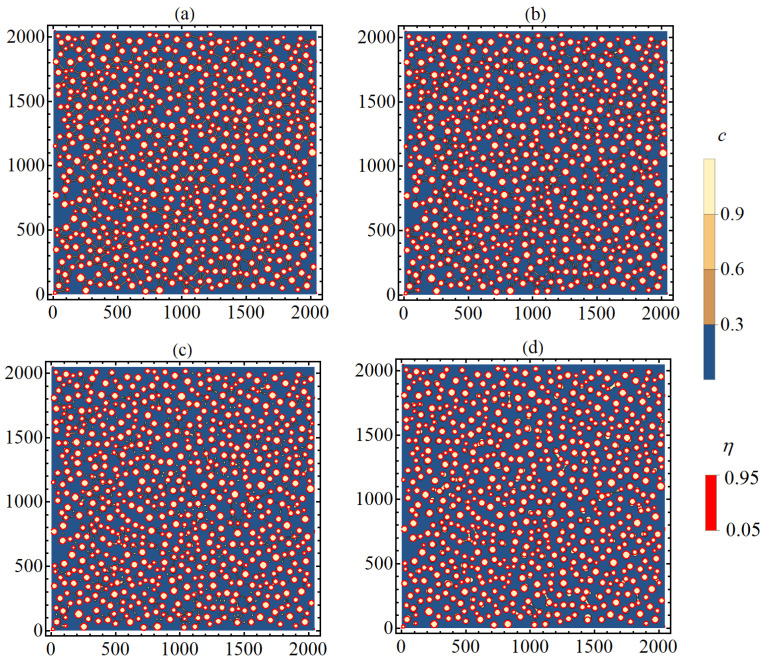
The results of simulation of filament rupture during thermal annealing of the initial structure (**a**) at different time points: t= 400 (**b**), t= 1000 (**c**), and t=6000 (**d**). The figures correspond to the system with the initial average particle size R=21 and quantity N=674.

An ensemble of particles with circular symmetry was formed as the initial condition. The initial distribution of particles was specified by solving the Cahn–Hillard Equation (5) for a regular solution with constant mobility and homogeneous interaction parameters in the region corresponding to unstable states, where the spinodal decomposition mechanism is realized (〈c〉=0.3, T=0.6, M=1, $\kappa_{c}=1.5$). Small particles with size R<1.5w=12 (*w* is the filament thickness) were removed from this region. An example of the obtained distribution of particles is shown by red contours in Figure 16a. For further simulation of the dynamics of filament rapture, two initial structures were used, obtained at different times t= 10,500 and t= 25,000, which differed in average size and number of particles equal to $\langle R_{0}\rangle =17,N_{0}=969$ and $\langle R_{0}\rangle =21,N_{0}=674$, respectively.

Then, filaments were randomly created between the particles with a probability density

$$f\left(r\right)∼\exp\left(-r/r_{0}\right),$$

where *r* is the distance between particles, $r_{0}=200$. The maximum filament length was limited from above by $r_{\max}=120$. All filaments crossing particles or other filaments were excluded. The initial distribution of the concentration field and the order parameter at the particle boundary were approximated using the hyperbolic tangent. The formed filaments had the same width equal to w=8. At the initial time, the concentrations in the particles, filaments, and matrix corresponded to the minima of the free energy density: c=0.9852 (P), c=0.042054 (M), and c=0.89038 (F). The temperature is taken to be equal to T=0.65.

Figure 16 shows the dynamics of the original two-dimensional structure containing particles interconnected by filaments (Figure 16a). In fact, the considered initial structure corresponds to a percolation cluster. If we assume that the transport of charge carriers occurs in regions with an increased concentration of *c*, then the initial percolation cluster provides charge transfer. At subsequent time points, the filaments are gradually destroyed (Figure 16c,d), and the original percolation cluster is fragmented into unbound parts. The assumed conductivity of the system under consideration vanishes.

**Figure 17:**
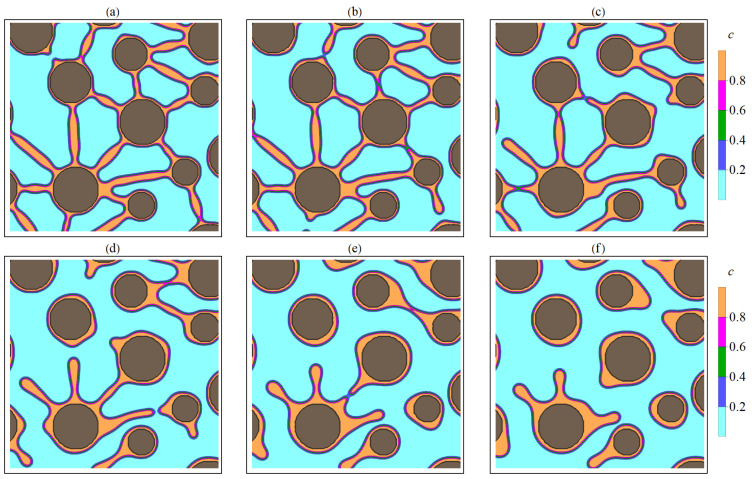
Filament break stages corresponding to different times *t*: (**a**)—120, (**b**)—220, (**c**)—400, (**d**)—600, (**e**)—1020, (**f**)—1400. The gray areas correspond to the crystallized phase (P). Light areas correspond to the matrix (M). Transition regions with increased *c* concentration correspond to filaments. The figures show areas with a size 200×200 (300<x<500,300<y<500).

**Figure 18:**
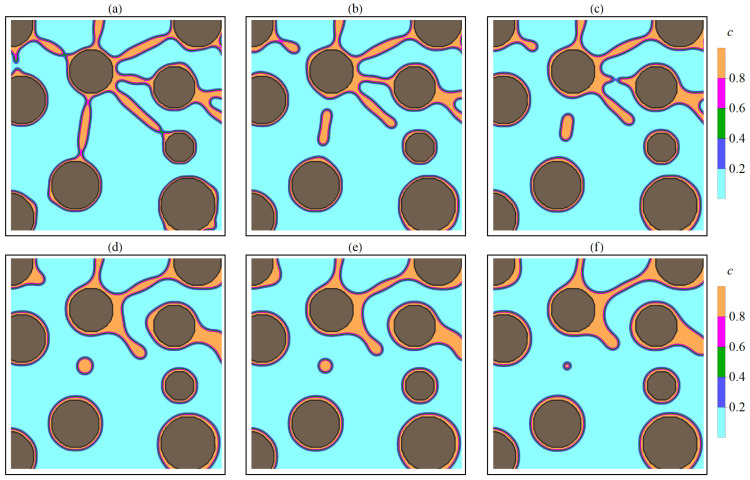
Filament break stages corresponding to different times *t*: (**a**)—220, (**b**)—400, (**c**)—600, (**d**)—1020, (**e**)—1400, (**f**)—2000. The gray areas correspond to the crystallized phase (P). Light areas correspond to the matrix (M). Transition regions with increased *c* concentration correspond to filaments. The figures show areas with a size 200×200 (1400<x<1600,900<y<1100).

Depending on filament length, two types of rupture were observed. Short filaments have a single point of discontinuity, which tends to be near the smaller particle. After the rupture, substance that made up the filament gradually moves to a larger particle, which leads to an increase in its size. For longer filaments, a break occurs in two places (near each of the interacting particles), while the remaining substance of the filament forms a small area with an increased concentration, which gradually tends to circular symmetry. Particles formed in the matrix during filament rapture are unstable and gradually dissolve, and the released substance is redistributed among larger particles due to diffusive transfer. Particles that have absorbed the substance of filaments also increase in size. Examples of breaking short and long filaments at different time points are shown in Figure 17 and Figure 18.

**Figure 19:**
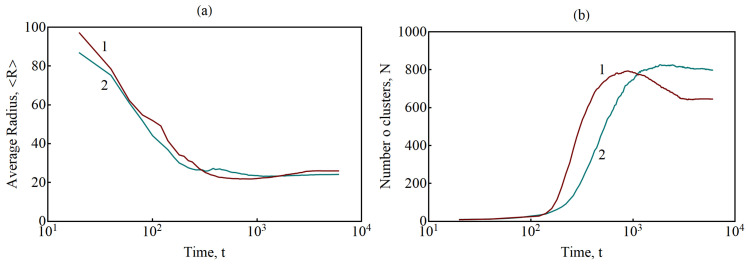
Dynamics of the average equivalent radius (**a**) and the number (**b**) of clusters formed during the rupture of filaments in the initial structure shown in Figure 16. The curves correspond to systems with different initial number of particles: 1—$\langle R_{0}\rangle =21,N_{0}=674$ and 2—$\langle R_{0}\rangle =17$, $N_{0}=969$.

Of interest is the dynamics of average characteristics at the rupture of filaments. The decay of a percolation cluster can be characterized by the number and size of isolated clusters or particles. The identification of unbound particles was carried out using the nearest neighbor method [42,43], which is widely used in the theory of cluster analysis. The bound particles were determined by the threshold value of the concentration $c_{th}>0.3$. The results of calculating the number of clusters and the average size are shown in Figure 19. In the decay process under consideration, the number of isolated clusters increases, while their average size decreases. The decrease in the number of isolated clusters and an increase in their size is due to the disappearance of unstable particles formed in the matrix as a result of the filaments dissolution (Figure 18). An increase in the number density of crystallized particles leads to a decrease in the average distance between them and, consequently, to a decrease in the fraction of long filaments, the decay of which leads to the formation of unstable particles in the matrix (Figure 18). This explains the presence of a longer section of the decrease in concentration for curve 1 in Figure 19b.

At the end of the simulation, the percolation cluster is completely destroyed, but the system still contains particles connected to each other by filaments (Figure 16d). As a rule, these filaments bind a small number of nearest particles (up to four). Thus, it can be assumed that the shortest filaments can correspond to a stable (metastable) state, the decay time of which can significantly exceed the ensemble average value. The proportion of short filaments increases with the increasing number density of crystallized particles in the original system, so the number of metastable filaments with longer decay time increases, which can be detected by the number of unbound particles ($N_{0}-N\left(t\right)$) at the end of simulation (Figure 19b).

## 6. Conclusions

Memristive percolation networks based on two-dimensional arrays of silver and gold nanoparticles have been synthesized and studied. Switching between memristive and capacitive states is investigated using cyclic voltammetry and step voltage excitation. The observed currents correspond to conductivity values less than one quantum of conductance. So, there is no fully formed percolation path in the system, and the conductivity remains controlled by tunnel junctions. At a constant voltage close to the breakdown voltage, the conductance grows according to a power law. The resulting memristive arrays of nanoparticles have the properties necessary for reservoir computing: they perform a non-linear transformation of input current signals, and they have a short-term memory (left to itself, the system relaxes to its original state due to the thermodynamic dissolution of filaments). The stability of the responses of the resulting arrays to the noise of the input signals requires additional research.

DFT-based simulation of the quantum contact of two nanoparticles confirmed wide distribution of conductance with variations in the position of single atoms in the gap between nanoparticles. Wide (power law) distribution of elementary conductances in an array is related to the Lévy statistics of tunneling conduction channels describing power-law evolution of current.

Based on the phase field theory, a model for the dissolution of filaments has been developed. The initial state of systems corresponds to a percolative state. During the dissolution of filaments, the percolation cluster breaks up into unbound regions. However, for a long time, the system still contains particles connected to each other by filaments. As a rule, these filaments bind a small number of nearest particles. It can be assumed that the shortest filaments correspond to a stable (metastable) state, the decay time of which significantly exceeds the ensemble average value.

## Figures and Tables

**Table 1:** Simulation parameters.

Parameter	Value
Size of the system	2048×2048
$\Omega^{P}$	1.2
$\Omega^{M}$	1.0
$g^{P}$	−0.25
$g^{M}$	0.1
$\kappa^{P}$	1.2
$\kappa^{M}$	1.0
$\kappa_{\eta }$	0.5
*W*	10

## Data Availability

The data presented in this study are available on request from the corresponding author.
